# “Self-testing sounds more private, rather than going to the clinic and everybody will find out”: Facilitators and barriers regarding HIV testing among men who purchase sex in Bali, Indonesia

**DOI:** 10.1371/journal.pone.0214987

**Published:** 2019-04-08

**Authors:** Luh Putu Lila Wulandari, Abby Ruddick, Rebecca Guy, John Kaldor

**Affiliations:** 1 The Kirby Institute, University of New South Wales, Sydney, NSW, Australia; 2 Department of Public Health and Preventive Medicine, Faculty of Medicine, Udayana University, Denpasar, Bali, Indonesia; 3 Independent Consultant, Denpasar, Bali, Indonesia; Simon Fraser University, CANADA

## Abstract

In many Asian countries, men who purchase sex account for the largest single network of people which often face elevated HIV risk in relation to the general population. However, high proportions of these men have never undertaken HIV testing. We assessed barriers to and facilitators of HIV testing among men who purchase sex in Indonesia, including the acceptability of HIV self-testing. A qualitative study was conducted during December 2016-January 2017 at fourteen sex-work venues and one voluntary HIV counselling and testing (VCT) clinic in Bali. Interviews were conducted with men who purchase sex exploring the men’s views on HIV testing. Data were examined using thematic analysis. Twenty-nine men participated in the study. The themes that emerged regarding the barriers to HIV testing included fear of potential shame, embarrassment, and confidentiality breach in accessing HIV testing; fear of social exclusion if the test result was positive; self-treatment and prevention; the distance to a clinic; time constraints; and fear of an invasive testing method. Factors that were seen as facilitating a test were the convenience of time and place; the provision of speedy results; and privacy. Participants expressed interest in HIV self-testing and preferred it to clinic-based testing due to the privacy and confidentiality of the results. The findings support the introduction of an HIV self-testing strategy among this group to improve access to HIV testing.

## Introduction

Between 8% and 35% of new reported HIV cases in almost all regions globally in 2017 were estimated to occur among men who purchase sex and sexual partners of high-risk groups [1], In many Asian countries, men who purchase sex represent the largest single network of people which often face elevated HIV risk in relation to the general population [2]. With a total population of men in Asia at more than 2.3 billion [3], estimates have ranged from 0.5% to 15% of the adult male population as having purchased sex [2] from female sex workers, among whom HIV prevalence was estimated at 5.2% in Asia in 2012 [4].

In addition to possible HIV exposure due to condom-less sex with female sex workers, other factors that might place men who purchase sex at an increased risk of HIV include having multiple sexual partners [5–9], sexual contact with men [10, 11], a high rate of sexually transmitted infections (STIs) that could increase HIV transmission [5–9, 12, 13], and injecting drug use [14–16]. Although some studies have reported a low HIV prevalence (0–0.5%) among men who purchase sex [17, 18], other studies have reported a high HIV prevalence, ranging from 5.7% to 26.5% [5, 9, 14, 19–22]. Systematic reviews of HIV prevalence among men who purchase sex in China and West and Central Africa found overall prevalence of 0.68% [23] and 7.3% [24] respectively.

In a number of countries, men who purchase sex have been found to have substantially higher HIV prevalence than men in the general population [19, 25–30]. Where condom use in sex work settings is at low or moderate levels, and HIV prevalence among sex working women is high, it is reasonable to assume that men who purchase sex are at higher risk and should therefore access HIV testing. For Indonesia, there have been very limited data available on HIV in this population, apart from surveys of occupational groups such as truck drivers and others [31], who may be considered to be more likely to purchase sex, due to mobility. The data of HIV prevalence among those occupational groups revealed the varying rates of HIV prevalence across provinces, i.e. between 0–0.8% [31]. The impetus for providing more opportunities for HIV testing among men who purchase sex is based on the potential risk of HIV acquisition and transmission in this population. Given the engagement of men who purchase sex with other key populations and the fact that this is a difficult-to-reach group that has not often been the focus of national and global prevention efforts, the need for HIV testing among this group is paramount.

HIV testing serves as an important gateway for preventing HIV transmission as well as for care and treatment. Acknowledging the importance of HIV testing, over the past few years the Indonesian government has intensified efforts to scale up access to testing facilities so as to reach higher risk populations, including men who purchase sex. Despite these efforts to scale-up testing access, testing coverage among men who purchase sex remains low. We conducted a survey of men who purchase sex in Bali in 2015 and found that only 8.1% of men who purchase sex had undertaken HIV testing [32], contrasting with other countries such as the US, Russia, Switzerland, Mexico, Hong Kong, Nepal, and China where HIV testing rates of between 16% and 74% have been reported [7, 8, 18, 33–36].

Globally, the UNAIDS coverage goal is for 90% of people living with HIV to be aware of their positive HIV status through an HIV test [37]. Recognising the difficulty of achieving this with clinic-based testing alone, the World Health Organization (WHO) released new guidelines in 2016 highlighting the importance of using self-testing to expand access to and coverage of HIV testing [38]. Several systematic reviews have reported high acceptability of this strategy among populations at risk [39–41]. There have also been many qualitative studies investigating the acceptability of HIV self-tests in the general population and other key populations but, to the best of our knowledge, none among men who purchase sex. Conducting qualitative research among men who purchase sex is important since they are at higher risk of HIV compared with the population overall, and may have particular views about testing modalities due to their behaviours being stigmatised and hidden. In the context of a broader research program focused on HIV testing among men who purchase sex through referral to nearby VCT clinics, we used in-depth interviews to explore barriers to, and facilitators of HIV testing among men who purchase sex in Bali, Indonesia, as well as the perceived acceptability of HIV self-testing.

### Setting

Indonesia is categorised as having a concentrated HIV epidemic; it is also one of the fastest growing HIV epidemics in Asia [42], with the current total number of people living with HIV infection estimated to be between 530,000–730,000 [43]. According to an Integrated Bio-Behavioural Surveillance (IBSS) conducted in 2013, the prevalence of HIV infection was highest in people who inject drugs (39.2%), transgender (7.4%), female sex workers (7.5%), men who have sex with men (12.8%), and prisoners (1.2%), with prevalence varying considerably across Indonesia [31].

As of 2017, about 242,699 HIV cases had been reported to the Indonesian Health Ministry since 1987, 66% of them among men [44]. Bali is one of the provinces in Indonesia that contributed the highest number of these cases, with heterosexual transmission being the second main mode, particularly in the context of transactional sex, which involves the exchange of sex for money, goods or favours [44]. As in many Asian countries, a distinction is made in Indonesia between so-called “direct”, or brothel-based female sex workers, whose main income is from selling sex, and “indirect” female sex workers, or non brothel-based female sex workers who work in massage parlours, bars, cafés and karaoke bars for their main income, but also offer sex services. It was estimated in 2017 that 5.2 million men in Indonesia bought sex from direct female sex workers [45].

## Materials and methods

The COREQ qualitative reporting guidelines [46] were used to guide the development of this report, with the information presented in accordance with the COREQ checklist. COREQ is a formal guideline aimed to improve the transparency of reporting of the data collection and analysis method used in qualitative studies. It contains a checklist of research team reflexivity, study design, and analysis and findings [46].

### Study design

Qualitative research was employed due to its interpretative approach to gaining an understanding of how people make sense of their lives [47]. Qualitative studies are the only means by which people’s attitudes to a new health technology can be properly understood, because they allow respondents to report their perceptions in detail at an individual level. They complement data from quantitative surveys, which have the benefit of being applicable at a population level, but can only provide binary information on predetermined items. This approach is suited to the current initial, exploratory study aimed to understand the barriers and facilitators to HIV testing, as well as the perceived acceptability of HIV self-testing, which are most likely to relate to people’s perceptions about HIV risk and how it might be mitigated. A number of earlier studies have used qualitative methods to understand perceptions on HIV testing and specifically HIV self-testing [48–50].

### Ethics information

Ethics approvals were obtained from the Research Ethics Committee of Udayana University/Sanglah Hospital, Indonesia and the Human Research Ethics Committees (HRECs) of the University of New South Wales, Australia.

### Timeline and participant selection

Data collection was undertaken between December 2016 and January 2017 in Denpasar, Bali. All sex venues formally recognised by the government for national and local health and IBBS surveillance purposes and one voluntary counselling and testing (VCT) clinic were selected.

The study population comprised men with a history of purchasing sex from female sex workers at any time. Purposive sampling was used to recruit participants based on the presumed likelihood of their providing detailed perspectives related to the research topic [51]. In order to include men with a range of views and experiences related to HIV prevention services, men who had sex with both brothel-based and non brothel-based female sex workers (such as those in massage parlours, cafés and bungalows) were recruited. In addition, to ensure we captured the views of men from different ethnic groups, we aimed to recruit men from diverse Indonesian ethnicities, which in Bali means primarily Balinese or Javanese men. We intended to recruit 20–30 participants, with the final sample size based on the achievement of data saturation, when no new meaningful information would likely be obtained from interviewing more participants [52].

### Procedure

Clients of brothel/non-brothel female sex workers were recruited with the assistance of fieldworkers from the Yayasan Kerti Praja (YKP), a non-governmental organisation (NGO) that provides HIV interventions for female sex workers in brothel and non-brothel areas. These outreach workers have been active at sex work venues for many years, and have been involved in various research activities such as local surveys or national surveillance. The fieldworkers approached men who presented at the brothels/massage parlours/bungalows/cafes during HIV interventions for female sex workers conducted by the NGO. These interventions included condom promotion and distribution to female sex workers, and referral for STI screening. The outreach workers did not know the potential participants, except for four participants who were clients at the time but were formerly employed at the brothels. In addition, men who presented at the YKP VCT clinic and were willing to participate in the study were recruited with assistance from the VCT clinic counsellor. These men were included in the study in order to understand the perspectives of those already undertaking HIV testing.

Semi-structured interviews [53] were conducted by LPLW (first author). Participants were interviewed in Indonesian. Interviews lasted about 30 minutes, were audio recorded, and conducted in private areas, guided by the participants’ preferences. Notes were also taken during the interviews. Before the interview, LPLW introduced herself as a PhD student who was studying health behaviours among men, and sought informed consent from those who were willing to be involved in the study.

An interview theme list [53] was used to guide the interview process. The questions covered in the interview theme list were developed from the findings of previous studies [32, 54, 55], and existing literature [56]. Subjects covered in the interview were: knowledge and perception of HIV and AIDS; sexual health experience; experiences with HIV testing services; preferences in the provision of HIV testing including self-testing; and stigma surrounding HIV. To improve the quality and validity of the findings, member checking was conducted at the time of the interview, that is, participants were asked to elaborate or clarify whether the researchers had captured correctly what the participants had said in the interviews [52, 57].

### Analysis

The recordings of the interviews were transcribed verbatim and were not translated into English as both researchers who undertook the analysis were fluent in Bahasa Indonesia. Thematic analysis was conducted to analyse the data [58]. Qualitative research analysis software NVivo 11 [59] was used to organise common topics and issues into particular codes, and to group the codes into particular themes.

To further develop the analysis and ensure the quality of the findings, two trained qualitative researchers who speak both English and Indonesian (LPLW and AR) assessed the transcripts for comparison of the emerging themes [60], and peer debriefing with colleagues was conducted [57].

## Results

About 50 men were approached, of whom 42% refused to participate. The majority of those who refused to take part were men who purchase sex from non brothel-based venues; and they expressed lack of time and concern about confidentiality as their main reasons for refusal. All those who agreed to participate in the study provided socio demographic characteristics before the interview. Of the 29 men interviewed, 24 were clients of brothel-based female sex workers. Sixteen were Balinese, and the rest Javanese. Four men had previously been involved as workers in brothels, but were no longer at the time of the interview. Only nine (31%) had undertaken HIV testing and of those, only four (13.8%) had undertaken HIV testing in the last year (Table 1).

**Table 1:** Study participants’ characteristics.

**Men’s characteristics**		**Accepted to participate (n = 29)**
**Brothel-based or non brothel-based sex workers’ clients**		
	Brothel-based sex workers’ clients	24 (83%)
	Non brothel-based sex workers’ clients	5 (17%)
**Ethnicity**		
	Balinese men	16 (55%)
	Non Balinese Men	13 (45%)
**Previous HIV testing**		
	Had undertaken HIV testing	9 (31%)
	Had never undertaken HIV testing	20 (69%)

Themes that emerged during the interviews in relation to HIV testing included fear of potential shame, embarrassment, and confidentiality breaches in accessing HIV testing; fear of social exclusion if the test result was positive; self-treatment and prevention; feeling healthy and a perceived low risk of contracting HIV; HIV as a life-threatening disease; and logistical barriers to testing. Participants expressed interest in HIV self-testing.

### Fear of potential shame, embarrassment, and confidentiality breaches in accessing HIV testing

This theme concerns the social consequences the participants perceived they would bear as a consequence of accessing HIV testing, including shame, embarrassment, and confidentiality breaches.

Participants understood that HIV testing was important for knowing one’s HIV status:

*For symptoms*, *we would never know it before we do the test. We would only know the status once we do the test. (yk10, Balinese, brothel-based sex worker‘s client)*

However, men expressed potential shame and embarrassment–over being seen at the clinic or seeking a test, or if there was a confidentiality breach of the testing result–due to the high degree of stigma attached to HIV.

*I will be embarrassed. People will question what I am doing at that clinic*. *(yk8, Balinese, non brothel-based sex worker‘s client)*

*I would be embarrassed*, *everybody will know the result. The confidentiality is not assured. We never know what will happen if we do the test when a lot of people are around*. *(yk8, Non Balinese, non brothel-based sex worker‘s client)*

*If I check my blood at the NGO*, *and my wife went there, according to the documents and the health recommendations, they’d mention it, directly, transparency. If anything happened, my name is in their records*. *(jg6, Balinese, brothel-based sex worker‘s client)*

### Fear of social exclusion if the test result was positive

In addition, participants expressed fear of social exclusion if the result was positive. They said they were concerned that were they tested positive, they would be excluded by family members, friends and people in their village, where gossip is a powerful social force.

*Never [talking about HIV with a friend]. I'm afraid that if I open up about my own disgrace*, *my friends will exclude me. I have been told that [if there are] people like that [who have HIV], [other people] will stay away from us. I know that… I am scared of that. They won’t hang out with me. That’s what I am scared of*. *(yk10, Balinese, brothel-based sex worker‘s client)*

*That’s people in Indonesia. Even if two people are just walking together, people will say that they’re dating*, *they will say they’re naughty, that is what people will think. In my place in Malang, [if] there is one person with HIV, people will stay away from them. Although as far as I know, [HIV] cannot be transmitted if we hang out together, don’t exchange saliva, not having sex, no blood from wounds. But, if you live in the village, still everybody will avoid you*. *If you get sick [with AIDS], everybody will avoid you. So it’s better to keep quiet. (cs21, Non Balinese, non brothel-based sex worker‘s client)*

*[Feeling ashamed towards] everybody… if we are positive… people will stay away from us*. *(cs25, Non Balinese*, *brothel-based sex worker’s client)*

*I am afraid of the results. It’s about the future, I’d rather not know the results. If it’s negative*, *that’s better, right, no problem. The thing is, if it’s positive, what if my family knows about it. We will feel ashamed. What is more, what if the whole village knows about it? They will isolate us. Family will feel ashamed too. That’s why it’s better to not know about it. Everyone will die anyway, we just don’t know when*. *(yk13, Non Balinese, brothel-based sex worker‘s client)*

The above quotes illustrate how strongly participants fear the social exclusion which might arise if they found they were HIV positive, and which might influence their decisions to take the test. The stigma around HIV, and the potential shame and embarrassment of being seen at the clinic, links with men’s preferences for self-treatment and testing, as mentioned below.

### “I have bought ampicillin for HIV”: Self-treatment and prevention

This theme describes an incorrect belief that antibiotics can prevent HIV and how men often use self-treatment for STIs and HIV. They said that they bought the treatment themselves when they had STI symptoms and believed it would prevent them from getting HIV or another STI.

*I had those symptoms, feeling hot around the genitals*, *and a little bit itchy. And the symptoms disappeared immediately after I took it [tyamicin]. (yk12, Non Balinese, brothel-based sex worker‘s client)*

*To prevent HIV, my friend told me*, *he said that if I ever had sex with them [female sex workers] without a condom, there is an antibiotic I need to take. I need to take it after I have a sex with the woman. (yk12, Non Balinese, brothel-based sex worker‘s client)*

*I bought the treatment myself when I had syphilis… got cured… no flare-ups until now. (bs16*, *Balinese, brothel-based sex worker‘s client)*

*I have bought ampicillin*, *tetra [tetracycline] [to prevent HIV]. Before having sex, I take one tablet [of ampicillin] to be safe. (bk22, Balinese, brothel-based sex worker‘s client)*

*If you wanna have sex with the woman [female sex workers] make sure you buy it [antibiotic] first [to prevent HIV]… all my friends in the village said so. (bk22*, *Balinese, brothel-based sex worker‘s client)*

*My friend said he once contracted an STI. He said it was syphilis… He didn’t go to the doctor. He didn’t go to the community health centres. He bought the antibiotic himself*. *I think there are a lot of men doing it [buying antibiotics themselves to treat STI rather than going to the clinic]. (yk7, Balinese, brothel-based sex worker‘s client)*

### Feeling healthy and a perceived low risk of contracting HIV

Men acknowledged that HIV can be transmitted through sexual contact, as expressed below:

*It is often transmitted through sexual contact, blood meets blood*, *that is what I know, blood meets blood. (yk13, Balinese, brothel-based sex worker‘s client)*

One man acknowledged the role of genital sores in transmission.

*Ehmm… it is transmitted through needles… well… if we have a genital ulcer and we have sex… is it? (bs15*, *Balinese, brothel-based sex worker‘s client)*

Due to the fact that they have taken medication as a preventive measure, men feel they are at low risk of contracting HIV even though they have had sexual contact with sex workers.

*[When I take thiamycin] I am sure I am healthy*, *that is what I usually do. Other people, I do not know. (lu3, Balinese, brothel-based sex worker‘s client)*

This perceived low risk of contracting HIV was bolstered by a belief among the men that they knew how to choose a “healthy” woman, chose the same woman at every visit, or purchased sex less frequently.

*I come here only during the day*. *At night I cannot see [the condition of the girl] clearly. If her eyes are white, nice, clean, then I know this is good. Likewise, if her skin is healthy, then I know she is healthy. If her skin does not look fresh, I do not want to ask her. Even if I was given a free service, I would not want to. (lu1, Balinese, brothel-based sex worker‘s client)*

*I guess [I'm not at risk of contracting HIV] because I do not often do it [buy sex]*. *(sb19, Balinese, non brothel-based sex worker‘s client)*

### HIV is a life-threatening disease

The concern that HIV/AIDS was a life-threatening disease led to a preference not to seek a test and to remain ignorant of HIV status.

*I'm afraid*. *It's [AIDS] a deadly disease… that is what I am scared of. I'm sure many people are also afraid to get an HIV test because of it. (cs28, Non Balinese, non brothel-based sex worker‘s client)*

*Actually I once invited him to do a test, but he said no*. *He said he’s better not knowing his status, even though he might die suddenly. (yk7, Balinese, brothel-based sex worker‘s client)*

*All I know is that the disease cannot be cured; probably will die right away*. *(yk9, Non Balinese, brothel-based sex worker‘s client)*

Logistical barriers were often cited to justify why men do not seek HIV testing or retesting.

### Logistical barriers to testing such as distance constraints, time constraints and fear of an invasive testing method

While some men simply did not know where to get tested, others were reluctant to go to the clinic due to distance and time constraints.

*If there's time*, *I might want to test… It's a problem of timing… I feel reluctant [when asked to go to a clinic]. Time becomes the problem. (sb19, Non Balinese, brothel-based sex worker‘s client)*

*I work*, *I could only go if it were on a holiday. (bk23, Balinese, brothel-based sex worker‘s client)*

*I don’t think I have time. I am a truck driver*, *rarely at home. (lu3, Balinese, brothel-based sex worker‘s client)*

Fear of an invasive testing method also emerged as a reason for the men’s reluctance to get an HIV test.

*I’m even afraid of injections. I have an experience when the needle was broken; when it was injected*, *it broke right away. (bk22, Balinese, brothel-based sex worker‘s client)*

*Probably get scared because it hurt [when being injected]. A blood test will hurt, they say. So when (I) get sick*, *just get the medicine [no injection]. (yk9, Non Balinese, brothel-based sex worker‘s client)*

### Facilitators of HIV testing

Participants suggested that were they given options such as where to do the test, at a time and place convenient for them, they would be willing to be tested.

One participant also said he would be willing to take the test if he did not need to wait too long for the results.

*My last test was about a year ago. But directly. I want to do a blood test*, *but I want the results to be instantly visible. If I have to wait for a long time for the results to come, I prefer not to have it. I will be worried. (bs15, Balinese, brothel-based sex worker‘s client)*

*People sometimes just don’t want to go [to the clinic]… getting treatment in there*, *well they just don’t wanna do that… Except that we do the test here [when we visit the brothels]… If we are asked to go to the clinic … it really depends on time availability. (lu3, Balinese, brothel-based sex worker‘s client)*

### Interest in HIV self-testing

When men in this study were asked about whether they would be willing to have an HIV test if they could do it themselves, they said they were willing to do so. When further probed on why this was the case, all said their preference for HIV self-testing over clinic-based testing was due to the simplicity, privacy and confidentiality of the results.

*Self-testing sounds more private*, *rather than going to the clinic and everybody will find out. (ma25, Non Balinese, brothel-based sex worker‘s client)*

*If there is one*, *I would prefer the one like a test pack [oral self-testing]. It’s more simple, and only we will know the result. If we go to a clinic, and people know it, I would be ashamed; people will be gossiping about me. (cs28, Non Balinese, non brothel-based sex worker‘s client)*

*If that is the case [self-testing]*, *we are more confident, it would be more private … It’s only me who will know it. Nobody else will know it. We just need to keep quiet … Just keep quiet and find the information on the treatment needed. Anyway, is there any treatment to cure the disease? (cs28, Non Balinese, non brothel-based sex worker‘s client)*

*It’s only me who knows [the result if I use self-testing]. (cs29, Non Balinese*, *non brothel-based sex worker‘s client)*

## Discussion

Stigma towards people living with HIV within health care facilities is significant in Indonesia [61–63], and engaging in commercial sex is viewed as socially unacceptable. Although participants acknowledged that HIV testing is important for knowing one’s HIV status, they were concerned about the social implications in accessing HIV testing at the clinic and receiving a positive result, and preferred a more confidential method of testing. Similar findings have emerged from studies among men who purchase sex in Guatemala [55] and South Africa [54], where confidentiality concerns hindered participants from having an HIV test, and in Tanzania, where confidentiality was a key factor in determining the choice of HIV testing strategy [64].

Confidentiality protection was an attractive feature of self-testing that most participants perceived in the study, saying that if they could choose, they would prefer self-testing to a test at a clinic; they would prefer to do the test themselves and perform the test at a time and place convenient to them to assure privacy and confidentiality. This is consistent with studies on the perceived benefits of self-testing in other populations, for example in South Africa [54], in the US [65], in Britain [66], and in Australia [67].

HIV self-testing also has the potential to overcome logistical barriers raised by men, such as distance from the clinic and time constraints. In other countries, a range of delivery methods for self-testing have been utilised, including online ordering,[68] purchasing from pharmacies [69], provision by women (both female sex workers and pregnant women) to male partners [70–75], household delivery [76], and vending machines [77]. These studies indicate that the efficiency of provision and saving of time are key motivators to using self-testing [65, 68, 78–80]. Fear of an invasive testing method also emerged as a reason for the men’s reluctance to get an HIV test. This issue can be resolved by HIV self-testing, especially modalities that make use of oral fluids [81]. A demonstration project using self-testing using oral fluids revealed a 6–7 times higher likelihood of testing uptake compared to conventional blood-based HIV testing at the clinic [82].

Our study reveals that the perceptions about HIV risk strongly influence men’s decisions about having an HIV test. Some believed they did not need to undertake HIV testing because they were at low risk of HIV. Other participants perceived HIV as a life-threatening disease and preferred not to seek the test and remain ignorant of their HIV status. Again, this finding is consistent with other studies, for example, from Zambia [83] and South Africa [54], both countries with a longer experience of HIV prevalence than Indonesia. To overcome these misconceptions, there needs to be a greater emphasis on education and community awareness. It is also imperative that the community understands the efficacy of modern HIV treatments in preventing disease progression and prolonging life, and that access to treatment can only be achieved if people know their HIV status–the sooner the better.

Other issues raised by men relate to knowledge about their risk of HIV and health implications of the virus. To overcome these issues, emphasis on education and community awareness might also be worth considering. Stigma also has a strong influence on men’s decisions to undertake HIV testing. Strategies to alleviate stigma and thereby reduce barriers to HIV testing include improving HIV-related knowledge, ensuring the provision of support to people living with HIV, and structural interventions such as laws to protect the rights of people living with HIV, or replacement of a counterproductive law [84]. In addition, a more robust public information campaign about the availability of free ARVs through the government health services, presenting HIV as a manageable chronic illness, would be beneficial.

Another interesting finding of the current study is that men preferred self-medication for STIs over going to a clinic. Men said they bought treatment themselves when they had STI symptoms, and believed it would protect them from both HIV and other STIs. Self-treatment among men who purchase sex, particularly by the use of antibiotics, has long been acknowledged in this setting [85, 86], and has also been observed in Cambodia. More than half of men who purchase sex who participated in a Cambodian study reported that they went to pharmacies for treatment for their last STI, compared with only 10% and 18% who went to private or public clinics, respectively [14]. Self-treatment is not ideal from a medical perspective, since most STIs do not have symptoms and over-treatment could facilitate antimicrobial resistance [87]. The preference for self-medication highlights the stigma around STIs, raising additional barriers to accessing clinics [88].

Some caution should be taken when interpreting the results of the current study. Although participants reported a preference for self-testing over clinic-based testing, we did not ask them specifically about their willingness to seek treatment at a clinic if they were positive in a self-test. Some studies internationally have reported suboptimal rates of linkage to care following self-testing [89], and in interviews, participants mentioned confidentiality concerns at the clinic, and fear of gossip within the community if they were seen going to the clinic. Another potential issue is that men were asked about HIV self-testing hypothetically, not having actual experience of it, so they may not have had a realistic view of its benefits or disadvantages. Also, the higher testing coverage (31%), compared with previous surveys (7–8.5%) among this population in Bali, may have arisen because some participants were recruited from a private VCT clinic. The refusal rate was high, which is not unexpected given the social stigma relating to buying sex. While the high refusal rate may have affected generalizability, the goal of a qualitative study is to provide a broad picture of views and perceptions, unlike quantitative studies which focus on generalization and prediction [90, 91].

Interpretation and input from the investigator is also an important part of a qualitative study. It is acknowledged that the researcher’s background will, to some extent, influence the data collection and analysis process [90]. The primary investigator, who conducted the interviews with participants, is a female Indonesian medical doctor trained in qualitative research who has been involved in various public health HIV interventions and research involving pregnant women and female sex workers in Bali, but had not previously been involved in providing care to men who purchase sex at the study sites. The first co-investigator is a female native English-speaking public health practitioner, with a PhD degree and anthropology background, who speaks fluent Indonesian and has been involved in various public health HIV interventions in Bali. The backgrounds of these researchers may have influenced how the data was collected and analysed.

## Conclusions

This study has revealed important perceptions related to structural and individual barriers to HIV self-testing in Bali. Men indicated that if they could choose, they would prefer a test that is confidential, so that only they would know the result. Men also expressed their interest in HIV self-testing, indicating the need for further research on the acceptability and feasibility of HIV self-testing among men who purchase sex. Future HIV programs should also continue to explicitly address misconceptions about HIV risk and disease progression, and to more vigorously emphasise the availability of treatment for HIV as a chronic condition.
